# Pain, Low Muscle Mass, and Exercise Participation After Myocardial Infarction: Determinants of Initial Exercise Testing at a Tertiary Center Serving a Rural Region

**DOI:** 10.3390/jcm15207951

**Published:** 2026-10-14

**Authors:** Jong-Joon Lee, Min-Cheol Joo, Eun-Sun Lee, Yeo-Bin Kim, Ji-Hee Kim

**Affiliations:** Department of Rehabilitation Medicine, Wonkwang University School of Medicine and Hospital, 895 Muwang-ro, Iksan-si 54538, Jeollabuk-do, Republic of Korea; kei02151@naver.com (J.-J.L.); jmc77@daum.net (M.-C.J.); dmstjs0802@naver.com (E.-S.L.); ybk1077@naver.com (Y.-B.K.)

**Keywords:** cardiac rehabilitation, exercise tolerance test, musculoskeletal pain, myocardial infarction, sarcopenia, skeletal muscle index

## Abstract

**Background/Objectives**: Musculoskeletal pain and balance impairments are common in patients referred for cardiac rehabilitation (CR), but their influence on early exercise participation and aerobic capacity remains unclear. This study examined whether pain and muscle mass are associated with initial exercise tolerance test (ETT) participation and VO_2_ peak. **Methods**: This retrospective, single-center cohort included 162 of 189 patients referred to CR after percutaneous coronary intervention for acute myocardial infarction; 105 who completed a symptom-limited cardiopulmonary exercise test were analyzed for VO_2_ peak. Pain intensity was assessed using a visual analog scale (VAS 0–10), and the skeletal muscle index (SMI) was measured using a bioelectrical impedance analysis. Determinants of ETT participation and VO_2_ peak were evaluated using multivariable logistic and linear regressions, respectively, with pain analyzed as a binary, three-category, and continuous variable. **Results**: More than half the patients reported musculoskeletal pain during physical activity, and 24.7% had balance problems. Patients with pain (VAS 1–10) had lower appendicular skeletal muscle mass and poorer functional status than pain-free patients. Among the 105 who completed the ETT, the pain group showed significantly lower workload-estimated metabolic equivalents [METs (Q)], respiratory exchange ratios, and peak minute ventilation and a shorter exercise time, but VO_2_ peak did not differ between the groups. ETT participation rates were similar across pain categories (63.4%, 67.2%, and 63.0% for VAS 0, 1–5, and 6–10, respectively). In the multivariable logistic regression, older age, hypertension, and low SMI scores were independently associated with reduced ETT participation. In the linear regression, age was the only significant predictor of VO_2_ peak; the SMI score showed a borderline association (*p* = 0.065), but pain intensity was not independently associated with VO_2_ peak. **Conclusions**: Musculoskeletal pain is prevalent among patients entering CR after myocardial infarction, and it is accompanied by low muscle mass and functional limitations. However, after adjustment, pain itself was not an independent determinant of ETT participation or VO_2_ peak. Low SMI scores were independently associated with reduced test participation and showed only a borderline association with aerobic capacity. These cross-sectional findings should be regarded as hypothesis-generating.

## 1. Introduction

Cardiac rehabilitation (CR) is a cornerstone of cardiovascular disease management, endorsed as a Class I recommendation in major clinical guidelines [1,2]. As a multidisciplinary program, CR integrates structured exercise training, nutritional counseling, smoking cessation, lifestyle modification, and psychological support—a comprehensive approach that promotes both functional recovery and delayed mortality.

Despite its established efficacy, participation in CR remains low worldwide. Global rates are estimated at 20–30% [3,4], with access particularly limited for elderly patients and those with comorbidities. Rural populations face additional barriers to CR participation, including poor accessibility to CR centers and challenges related to attendance and adherence [5,6]. In Korea, CR has been covered by the National Health Insurance since 2017, but only 5.8% of patients with acute coronary syndrome actually participate [7,8]. Limited facility access and lack of patient awareness are thought to contribute to this gap, leaving many patients unable to benefit from a proven intervention. In that nationwide analysis, cardiac rehabilitation was delivered predominantly at tertiary hospitals (82.6%), and patients living in urban areas were more likely to attend than those in rural areas [8], indicating that access barriers such as distance operate largely before patients reach a CR center. Once patients have been referred, however, patient-level factors may still determine whether they engage in the first step of rehabilitation, and patients who decline or defer initial testing have few alternative routes back into a structured program. Our hospital is a tertiary CR center whose catchment area includes extensive rural districts, which allowed us to examine these patient-level factors in a cohort in which access barriers had, at least in part, already been overcome.

A potentially important but underexplored barrier is musculoskeletal dysfunction. Standard CR programs typically follow uniform exercise protocols and offer little accommodation for individual physical limitations. Musculoskeletal pain—particularly low back pain—is common in patients entering CR and can promote exercise avoidance, acting as a significant deterrent to CR participation [9,10]. Musculoskeletal limitations have also been examined as a barrier to CR enrollment and participation [11], and reduced physical activity following myocardial infarction can lead to muscle weakness and restricted joint mobility, which further compromise rehabilitation effectiveness and predispose patients to secondary complications. Despite those clinical observations, the quantitative effects of musculoskeletal limitations on CR program participation and objective cardiopulmonary function remain poorly characterized. Existing reports have largely documented the presence of these barriers, describing their prevalence in rehabilitation cohorts or their association with self-reported adherence, without relating them either to objectively measured cardiopulmonary function or to concurrently quantified skeletal muscle mass [9,10,11]. That distinction is clinically important, because low muscle mass is common in cardiovascular populations and is itself an established determinant of functional decline and reduced aerobic capacity [12,13,14]. Pain and low muscle mass may therefore constrain rehabilitation through different mechanisms, one behavioral and one structural, and separating them requires that participation and measured exercise capacity be treated as distinct outcomes.

This study aimed to determine the prevalence of musculoskeletal pain among patients referred for CR after myocardial infarction and evaluate whether pain intensity and skeletal muscle mass are independent predictors of initial exercise tolerance test (ETT) participation and peak oxygen uptake (VO_2_ peak). By assessing musculoskeletal pain and body composition at the same referral visit, and by modeling participation in the initial test and measured VO_2_ peak as two separate regression outcomes, we sought to establish whether pain limits early rehabilitation engagement in its own right or mainly identifies patients in whom muscle mass is already reduced.

## 2. Materials and Methods

### 2.1. Patients

This retrospective, single-center study analyzed data from 198 patients who were referred for CR after percutaneous coronary intervention (PCI) for acute myocardial infarction (ST-segment elevation or non–ST-segment elevation myocardial infarction) at a single tertiary care institution in Iksan, Republic of Korea, between February and October 2024; patients who underwent PCI for chronic coronary syndrome were not part of this cohort. Two patients who died during hospitalization and seven who could not complete the survey were excluded, leaving 189 patients who completed standardized screening questionnaires for musculoskeletal pain and balance problems at referral. Body composition, including the skeletal muscle index (SMI), was assessed in a subset of the cohort using bioelectrical impedance analysis. Bioelectrical impedance analysis was unavailable for 27 of these 189 patients, who were accordingly excluded from all analyses involving body composition. In total, 162 patients (135 males and 27 females) were included in the primary analysis, and the 105 who completed the initial ETT were included in the analyses of exercise capacity. The screening questionnaire was completed at the inpatient CR consultation, a median of 1 (IQR 0–2) days after PCI (median length of stay 3 [IQR 3–5] days), and the predictors were not reassessed during the course of rehabilitation. On the basis of the residential address recorded in the medical records, place of residence was classified according to the Korean administrative system as a metropolitan city, another city (a dong [urban neighborhood] of a city), or rural (an eup or myeon [town or township] or a county [gun]). Only 2 patients (1.2%) lived in a metropolitan city, 61 (37.7%) resided in rural areas, and 87 (53.7%) lived outside Iksan, the city in which the hospital is located (Table 1).

This study was approved by the Institutional Review Board of Wonkwang University Hospital, Iksan, Republic of Korea (IRB approval number: 2024-11-039; approval date: 23 December 2024) and conducted in accordance with the Declaration of Helsinki. Patient data were kept confidential and used only for research purposes. Clinical variables were collected from the electronic medical record system: age, sex, type of acute myocardial infarction, left ventricular ejection fraction (LVEF), body mass index (BMI), SMI, the major comorbidities of hypertension, diabetes, and smoking status, years of education, medications at discharge (beta-blockers and statins), peak troponin T during the index admission (high-sensitivity assay, ng/L), the number of diseased vessels on coronary angiography, and residential address.

### 2.2. Assessment of Musculoskeletal Pain, Balance, and Functional Capacity

Upon referral to CR, all patients completed a structured questionnaire assessing musculoskeletal pain and balance problems. Pain intensity was measured using a visual analog scale (VAS) ranging from 0 (no pain) to 10 (worst imaginable pain) and analyzed in three ways: as a continuous score; in three categories, namely no pain (VAS 0), mild-to-moderate pain (VAS 1–5), and severe pain (VAS 6–10); and as a binary variable (VAS 0 vs. VAS 1–10). The binary contrast was used for the primary group comparisons because the purpose of screening at referral was to identify any pain that might require modification of exercise testing or training; the three-category and continuous analyses were used to examine dose–response relationships. Functional limitation due to pain during moderate-intensity physical activity (e.g., walking, cycling) was categorized as none, mild, moderate, or severe.

Balance disturbance was assessed using a self-administered questionnaire addressing instability during daily activities or exercise and a history of balance-related falls. Balance disturbance was dichotomized as present or absent. The questionnaire also asked patients to indicate previously diagnosed conditions on a checklist with space for free text; a chronic musculoskeletal disorder was considered present if a spinal disorder (herniated disc, spinal stenosis, or vertebral fracture), arthritis (degenerative or inflammatory), osteoporosis, or another chronic musculoskeletal condition was reported.

Functional capacity was assessed using the Korean Activity Scale/Index (KASI), a self-administered questionnaire adapted from the Duke Activity Status Index (DASI) for Korean patients. It estimates functional capacity based on the ability to perform specific daily activities, with higher scores indicating better functional status; the KASI was developed and validated against treadmill exercise time in Korean patients with cardiovascular disease [15], and its parent instrument, the DASI, correlates well with measured peak oxygen uptake [16].

### 2.3. Exercise Tolerance Test

An ETT was scheduled for all patients at the first outpatient CR visit after discharge to assess cardiopulmonary function; the 105 patients who completed it (a median of 16 [IQR 13–20] days after PCI) were included in the exercise capacity analyses. The test was performed on a treadmill (Q-Stress TM55, Mortara Instrument Inc., Milwaukee, WI, USA) with continuous respiratory gas analysis (TrueOne^®^ 2400, ParvoMedics, Salt Lake City, UT, USA). Blood pressure and heart rate were monitored using an automated system (247BP, SunTech Medical, Morrisville, NC, USA), and 12-lead electrocardiography (Quinton Q-Stress, Mortara Instrument Inc., Milwaukee, WI, USA) was recorded before, during, and after the test. When the attending physician judged a treadmill test to be unsafe or infeasible on the day of assessment (e.g., because of musculoskeletal pain or gait impairment, a neurological or cognitive disorder, or exertional symptoms), a 6-min walk test was performed instead; these patients, together with those who did not undergo any exercise test, were classified as ETT non-participants.

The participants completed a symptom-limited exercise test using a physician-modified Bruce protocol in accordance with American Association of Cardiovascular and Pulmonary Rehabilitation guidelines. The test was terminated upon the development of symptoms such as chest pain, severe leg pain, or dizziness or at the participant’s request.

The following parameters were measured: maximal systolic and diastolic blood pressure, maximal heart rate, VO_2_ peak, peak minute ventilation, metabolic equivalents (METs), respiratory exchange ratio (RER), and total exercise duration. METs were obtained in two ways: METs (Q) were estimated by the exercise-testing system from the treadmill speed and grade of the final stage attained, whereas METs (M) were calculated from directly measured VO_2_ peak (VO_2_ peak divided by 3.5 mL·kg^−1^·min^−1^). Cardiopulmonary exercise capacity was compared between patients without pain (VAS 0) and those with pain (VAS 1–10), and across the three pain categories, with VO_2_ peak and METs as the primary outcome measures.

### 2.4. Bioelectrical Impedance Analysis

Body composition was assessed using a bioelectrical impedance analysis (InBody 370^®^; InBody Co., Seoul, Republic of Korea), which applies electrical currents at multiple frequencies (5, 50, and 250 kHz); measurements were obtained during the index admission in 155 patients (95.7%; median 1 [IQR 1–2] day after PCI) and at the first outpatient visit in the remaining 7. Appendicular skeletal muscle mass (ASM) was defined as the sum of upper and lower extremity muscle mass, and the SMI was calculated as the ASM divided by height squared (kg/m^2^). The SMI score was used as the primary indicator of muscle status in the multivariable analyses.

### 2.5. Statistical Analysis

Participants were divided into two groups by pain status: no pain (VAS 0) and pain (VAS 1–10). Continuous variables are presented as the mean ± standard deviation, and categorical variables are presented as counts and percentages. The normality of continuous variables was assessed with the Shapiro–Wilk test in each group. Normally distributed variables were compared with independent-samples *t*-tests (or one-way analysis of variance across three groups), and non-normally distributed variables with the Mann–Whitney U test (or the Kruskal–Wallis test). Categorical variables were compared with the chi-square test or, when any expected cell count was below 5, with Fisher’s exact test (2 × 2 tables) or the Fisher–Freeman–Halton exact test (larger tables). The same methods were used to compare patients across the three pain categories, men and women, and ETT participants and non-participants.

A multivariable logistic regression was used to identify factors associated with ETT participation (1 = participated) among the 162 patients with complete body composition data. Covariates were prespecified on the basis of clinical relevance and prior literature rather than selected by univariable screening or stepwise procedures: sex, age, pain (VAS ≥ 1), diabetes mellitus, hypertension, and LVEF as demographic and clinical factors, and SMI as the measure of muscle status. ASM was not entered together with SMI because the two were strongly correlated (r = 0.82). With 57 non-participants, the model had 8.1 events per variable. A linear regression was used to identify the determinants of VO_2_ peak among the 105 patients who completed the ETT, with pain entered as a continuous variable; the number of covariates was limited to five (sex, age, pain intensity, LVEF, and SMI) given the sample size. Multicollinearity was assessed with variance inflation factors, all of which were below 2. In sensitivity analyses, pain was entered as a continuous score and in three categories, and balance problems, self-reported musculoskeletal disorders, and years of education (as a proxy for socioeconomic status) were added to each model, individually and together. In further sensitivity analyses, beta-blocker use at discharge, log-transformed peak troponin T, multivessel disease (≥2 diseased vessels), and rural residence, abstracted from the medical records, were added individually and together, and the models were repeated in patients whose body composition was measured during the index admission. Because all eligible patients during the study period were included, no a priori sample size calculation was performed; post hoc power was estimated by simulation (2000 replicates) for the logistic model and from the noncentral F distribution for the linear model. Analyses were performed using SPSS version 27 (IBM Corp., Armonk, NY, USA); exact tests, sensitivity analyses, and power calculations were performed using Python 3.13 with SciPy 1.18 and statsmodels 0.15, with statistical significance set at *p* < 0.05 (two-sided).

## 3. Results

### 3.1. Prevalence of Musculoskeletal Pain and Balance Problems (n = 162)

Among the 162 patients included in the primary analysis (Figure 1; mean age 65.9 ± 10.9 years; 83.3% male), the females were significantly older and had lower SMI and education levels than the males (all *p* < 0.05; Table 1). Musculoskeletal pain was prevalent, with more than 50% reporting pain during physical activity and 24.7% reporting balance problems (Table 2). Women more often reported pain (81.5% vs. 51.1%, *p* = 0.004) and pain medication use (37.0% vs. 19.3%, *p* = 0.043) than men, whereas the prevalence of balance problems did not differ by sex (25.9% vs. 24.4%, *p* = 0.871). Sixty-six patients (40.7%) reported a previously diagnosed chronic musculoskeletal disorder, most commonly a spinal disorder (*n* = 46; herniated disc, 25; spinal stenosis, 24), followed by arthritis (*n* = 22) and osteoporosis (*n* = 9).

Patients with pain (VAS 1–10) were more likely to be female (*p* = 0.004) and have lower ASM (*p* = 0.022) and KASI scores (*p* = 0.027) than pain-free patients. Age, BMI, and LVEF did not differ significantly between the groups (Table 3). Diabetes was less frequent in the pain group (23.1% vs. 60.6%, *p* < 0.001), whereas a history of musculoskeletal disorders was more frequent (52.7% vs. 25.4%, *p* < 0.001). Beta-blocker use at discharge, peak troponin T, and multivessel disease did not differ between the pain groups. When pain was analyzed in three categories (Supplementary Table S1), balance problems (12.7%, 21.9%, and 63.0%), self-reported exercise limitation (18.3%, 67.2%, and 74.1%), and a history of musculoskeletal disorders (25.4%, 35.9%, and 92.6%) increased across the VAS 0, 1–5, and 6–10 groups (all *p* < 0.001).

### 3.2. Effects of Musculoskeletal Pain on Initial Exercise Capacity (n = 105)

Among the 105 patients who completed the initial ETT, those with musculoskeletal pain (VAS 1–10) showed significantly lower METs (Q), VEmax, and RER and a shorter exercise time than the pain-free patients (Table 4). However, VO_2_ peak did not differ significantly between the groups. Notably, workload-estimated METs (Q) was reduced in the pain group, whereas both measured VO_2_ peak and its corresponding METs (M) were preserved.

Across the three pain categories (Supplementary Table S2), exercise time and METs (Q) were numerically lower in both pain groups than in the pain-free group (*p* = 0.056 and *p* = 0.066, respectively), without a meaningful further decrease from mild-to-moderate to severe pain, and VO_2_ peak did not differ (22.8 ± 6.5, 20.5 ± 6.2, and 21.2 ± 6.5 mL/kg/min; *p* = 0.255).

### 3.3. Determinants of Initial ETT Participation (n = 162)

Of the 57 patients who did not undergo the initial ETT, 26 underwent a 6-min walk test instead (median distance 240 m, IQR 126–360 m), and 31 underwent no exercise test. According to the medical records, the 6-min walk test was chosen because of musculoskeletal pain or gait impairment in 19 patients, a neurological or cognitive disorder in 4, and exertional symptoms in 3. Of the 31 patients without an exercise test, 23 did not attend the outpatient assessment and had no documented reason, 3 cited distance or were transferred to another hospital, 2 declined, 2 were in poor general condition, and 1 cited personal reasons. Compared with participants, non-participants were older (71.2 ± 10.7 vs. 63.1 ± 9.6 years), more often had hypertension (86.0% vs. 57.1%), and had lower SMI (7.51 ± 1.10 vs. 8.20 ± 1.10 kg/m^2^), KASI scores (51.0 ± 22.4 vs. 65.6 ± 17.3), and educational attainment (9.7 ± 4.6 vs. 11.6 ± 3.8 years) (all *p* < 0.01). In contrast, the prevalence of pain (54.4% vs. 57.1%, *p* = 0.736), pain intensity (*p* = 0.814), self-reported exercise limitation (*p* = 0.678), balance problems (*p* = 0.462), pain medication use (*p* = 0.792), and musculoskeletal disorders (*p* = 0.552) did not differ, nor did beta-blocker use at discharge (*p* = 0.245), peak troponin T (*p* = 0.614), or multivessel disease (*p* = 0.521) (Supplementary Table S4), and ETT participation did not differ significantly between rural and urban residents (57.4% vs. 69.3%, *p* = 0.123). The 26 patients assessed with the 6-min walk test were the oldest subgroup (75.1 ± 8.3 years) and had the lowest SMI (7.1 ± 1.1 kg/m^2^) and KASI scores (45.8 ± 22.1) and the highest prevalence of balance problems (42%) and musculoskeletal disorders (58%).

In the multivariable logistic regression, older age (OR 0.940 per year, 95% CI 0.902–0.980, *p* = 0.004) and hypertension (OR 0.226, 95% CI 0.087–0.587, *p* = 0.002) were independently associated with reduced ETT participation, whereas higher SMI was associated with greater participation (OR 1.795 per 1 kg/m^2^, 95% CI 1.131–2.847, *p* = 0.013); that is, lower SMI was independently associated with reduced ETT participation (Table 5). Musculoskeletal pain was not an independent predictor after adjustment for body composition and clinical factors. These results were materially unchanged when pain was entered as a continuous score (OR 0.943 per point, *p* = 0.446) or in three categories; when balance problems, self-reported musculoskeletal disorders, years of education, beta-blocker use at discharge, peak troponin T, multivessel disease, or rural residence were added individually or together; and when the analysis was restricted to the 155 patients whose body composition was measured during the index admission (SMI OR 1.66–1.81, all *p* ≤ 0.036). None of these additional variables was associated with participation; rural residence showed a nonsignificant trend (OR 0.54, 95% CI 0.25–1.18, *p* = 0.121) (Supplementary Table S3). The post hoc power of the logistic model to detect the observed SMI association was 0.74.

### 3.4. Determinants of Aerobic Capacity (VO_2_ Peak)

Among the 105 patients who completed the ETT, the multivariable linear regression identified age as the only significant determinant of VO_2_ peak (*p* < 0.001), with older age associated with lower values. The SMI score showed a borderline association (*p* = 0.065). Sex, ejection fraction, and pain intensity were not significantly associated with VO_2_ peak (Table 6). In the sensitivity analyses, the SMI estimate ranged from 0.92 to 1.49 (*p* = 0.032–0.139) and reached *p* < 0.05 only when rural residence was added or when the analysis was restricted to patients whose body composition was measured during the index admission, and a self-reported history of musculoskeletal disorders showed a borderline inverse association with VO_2_ peak (B −2.37 to −2.42, *p* = 0.049–0.051); the other added variables were not associated with VO_2_ peak (Supplementary Table S3). The post hoc power to detect the observed SMI association (partial f^2^ = 0.035) was 0.48; approximately 225 patients would have been required for 80% power.

## 4. Discussion

This study demonstrates that musculoskeletal pain is highly prevalent in patients referred for CR after myocardial infarction and frequently clusters with balance problems and lower appendicular skeletal muscle mass. Although pain was associated with lower effort-dependent exercise measures—including lower METs (Q), VEmax, and RER and a shorter exercise time—it was not an independent determinant of peak aerobic capacity (VO_2_ peak). Instead, participation in the initial ETT was primarily associated with age, hypertension status, and the SMI score, a height-adjusted index that is less influenced by sex and body size than absolute ASM. Pain was also not associated with test participation, whether analyzed as present or absent, in three categories, or as a continuous score. These cross-sectional findings are consistent with the possibility that musculoskeletal pain mainly affects effort-dependent performance rather than imposing a direct cardiopulmonary limitation, although the design does not allow causal inference.

Musculoskeletal pain is highly prevalent among patients with coronary artery disease and has been consistently associated with low physical activity, impaired functional capacity, and poor adherence to CR programs. Previous studies have identified pain-related fear of movement and exercise avoidance as significant barriers to participation in structured exercise interventions [17,18,19]. In our study, patients with musculoskeletal pain demonstrated lower KASI scores, more balance problems, and greater self-reported exercise limitation at baseline, compared with those who were pain free.

Patients with musculoskeletal pain exhibited significantly lower VEmax and RER and a shorter exercise time, but VO_2_ peak remained comparable between groups. VEmax and RER are key indicators of exercise effort and physiological response [20], and in the absence of maximal voluntary ventilation (MVV) data, reduced VEmax should be interpreted as reflecting attenuated effort rather than true ventilatory failure [21,22]. The two metabolic-equivalent estimates showed the same pattern: workload-estimated METs (Q) was significantly lower in the pain group, whereas measured VO_2_ peak and its metric-unit equivalent, METs (M), were preserved. Because METs (Q) is derived from the treadmill workload attained, and therefore reflects how long and how hard a patient is willing to walk, the divergence between estimated and measured values is what would be expected if pain curtailed volitional effort rather than aerobic capacity itself. This dissociation suggests that pain may primarily influence effort-dependent aspects of performance [23]. Notably, these differences did not increase from mild-to-moderate to severe pain, and no dose–response relationship with VO_2_ peak or test participation was observed.

In the adjusted model, pain intensity was not associated with VO_2_ peak, suggesting that, although musculoskeletal pain may limit maximal volitional effort, it was not an independent correlate of aerobic capacity in this cohort. Age was the only significant determinant of VO_2_ peak, and the association with SMI was borderline (*p* = 0.065) and should be interpreted cautiously given the limited power of this model [24,25,26]. One possible explanation, which our cross-sectional data cannot test, is that pain-related inactivity contributes to muscle loss over time, which in turn may limit exercise participation and capacity [12,24,27,28]; prospective studies are needed to examine this sequence and whether addressing pain and muscle loss improves CR outcomes [19,23,29,30].

Sarcopenia, characterized by reduced muscle mass and function, has been consistently linked to impaired physical performance and adverse outcomes, including disability, hospitalization, and mortality [12,31]. In patients with cardiovascular disease and heart failure, sarcopenia or muscle wasting has been reported in approximately 20–25% of patients, depending on the clinical setting and diagnostic criteria, and it is a potent predictor of adverse outcomes and reduced VO_2_ peak [13,14].

From a mechanistic perspective, sarcopenia involves a chronic inflammatory state with elevated pro-inflammatory cytokines such as TNF-alpha and IL-6 [24]. This systemic inflammation can exacerbate cardiovascular vulnerability and contribute to diminished exercise capacity. Mitochondrial dysfunction and impaired oxidative metabolism in skeletal muscle are also major determinants of reduced VO_2_ peak, independent of the left ventricular ejection fraction [32]. These skeletal muscle abnormalities contribute substantially to exercise intolerance, independent of cardiac function [32,33].

The benefits of CR extend beyond hemodynamic improvement to include positive effects on peripheral muscle metabolism and function [34]. In our study, the SMI score was independently associated with ETT participation and showed only a borderline association with VO_2_ peak after adjustment for clinical variables, aligning with prior work linking low muscle mass and frailty to reduced exercise capacity in elderly cardiovascular cohorts [14,33,35,36]. A prior study in elderly patients with coronary artery disease showed that structured CR participation resulted in dose-dependent improvements in SMI scores and VO_2_max over three months [37]. Those findings suggest that insufficient CR participation may accelerate muscle loss, whereas adequately dosed exercise interventions may help reverse it and improve functional outcomes [37,38,39].

Low muscle mass has been proposed as a mediator between pain-related inactivity and reduced functional capacity, although its role in limiting aerobic performance warrants prospective investigation. Musculoskeletal pain might accelerate this cycle by promoting inactivity and contributing indirectly to secondary muscle loss [38,39,40]; whether addressing these barriers early in CR prevents functional decline remains to be tested.

Current ESC and ACC/AHA guidelines strongly recommend exercise-based CR as a Class I intervention after myocardial infarction and revascularization. The established efficacy of CR—including a 26% reduction in cardiovascular mortality in meta-analyses [41] and a 38.8% reduction in the 3-year hazard ratio for all-cause death in Korean data [8]—underscores the importance of increasing participation. Nonetheless global participation rates remain suboptimal, often less than 50% of eligible patients, with even lower uptake in elderly and comorbid populations. Recent position papers emphasize that improving accessibility, addressing frailty, and tailoring programs to individual functional limitations are critical to closing this gap [42].

Our findings raise the possibility that assessing muscle mass and musculoskeletal pain at CR entry could help identify patients at risk of not engaging in initial exercise testing. Because low SMI was independently associated with non-participation, muscle-targeted strategies may merit evaluation in this group. Several recent reviews and trials have shown that combining aerobic training with resistance exercise and nutritional support can significantly improve muscle mass, VO_2_ peak, and functional outcomes in older adults and in populations with cardiovascular disease [39,43]. Whether such interventions improve test participation in patients entering CR with musculoskeletal complaints, low SMI scores, or both has not been established and requires prospective trials.

Balance problems were significantly more frequent in patients with pain than in those without pain, consistent with evidence that musculoskeletal pain, reduced proprioception, and lower extremity weakness interact to increase the fall risk in elderly cardiovascular patients. Previous studies using computerized posturography have demonstrated impaired static and dynamic balance in cardiac patients, compared with healthy controls [44]. Balance problems were not included in the prespecified models because of their conceptual overlap with pain, with which they were moderately correlated (r = 0.36 with VAS score). However, collinearity was low (all variance inflation factors < 2), and when balance problems were added in sensitivity analyses, they were associated with neither ETT participation (OR 0.73, *p* = 0.485) nor VO_2_ peak (*p* = 0.899), and the other estimates were unchanged (Supplementary Table S3). Because balance was assessed with a single self-reported item, objective balance measures would be needed to determine its independent contribution. Incorporating balance assessments and targeted balance training into CR may help to mitigate fall risks and improve exercise confidence.

The strengths of this study include our systematic assessment of musculoskeletal pain and balance problems at CR referral, measurement of body composition at referral and of exercise capacity at the first outpatient visit, the use of both logistic and linear regressions to identify predictors of exercise participation and VO_2_ peak separately, and sensitivity analyses examining alternative pain categorizations and additional covariates.

This study also has several limitations. The retrospective, single-center design precludes conclusions about cause-and-effect relationships among musculoskeletal pain, muscle mass, exercise participation, and aerobic capacity. Although the multivariable regression adjusted for relevant covariates, residual confounding is likely: frailty, self-rated health, socioeconomic status other than education, and baseline physical activity were not available, and infarct severity was represented by LVEF, infarct type, peak troponin T, and the number of diseased vessels only; adjustment for discharge beta-blocker use, peak troponin T, multivessel disease, and rural residence did not change the findings. Musculoskeletal disorders were self-reported on the screening questionnaire rather than verified diagnoses. Skeletal muscle mass was assessed using bioelectrical impedance analysis, which is practical and widely used but can show measurement variability in patients with altered hydration status. Pain, balance, and functional capacity were assessed only once, at referral and shortly after the acute event; symptoms related to the myocardial infarction itself may have influenced these reports, and these factors may change substantially during recovery. Additionally, ETT participation was itself an outcome, and only 105 of 162 patients completed the initial ETT; because non-participants were older and had lower muscle mass and functional capacity, the VO_2_ peak analysis was restricted to a selected, healthier subgroup. Reasons for non-participation were abstracted retrospectively from the medical records and were not documented for 23 patients who did not attend the outpatient assessment. No a priori sample size calculation was performed; the logistic model had 8.1 events per variable, and the linear model had limited power (0.48) to detect the observed SMI association, so the borderline SMI–VO_2_ peak association should be regarded as exploratory. Furthermore, muscle strength and physical performance were not assessed, so sarcopenia could not be formally diagnosed according to consensus criteria; SMI was used as a measure of muscle mass, and we therefore refer to low muscle mass rather than sarcopenia when describing our findings. Relatedly, body composition was available for 162 of the 189 screened patients, and the 27 without bioelectrical impedance data had to be excluded; because these measurements were obtained during routine care rather than mandated by protocol, the analyzed subset may not be fully representative of all patients referred after myocardial infarction, and selection bias cannot be excluded. The study was conducted at a single tertiary hospital whose catchment area includes rural districts; rurality was characterized only by administrative place of residence, and travel distance to the hospital was not measured, so the findings may not generalize to other settings. Finally, this analysis focused on baseline exercise testing at referral; longitudinal changes in exercise performance and long-term outcomes such as rehospitalization and mortality were not assessed.

In summary, musculoskeletal pain was common at CR entry and was associated with lower effort-dependent exercise measures, but it was not associated with participation in the initial ETT or with VO_2_ peak after adjustment, whereas low muscle mass was independently associated with non-participation. Screening for musculoskeletal pain, low muscle mass, and balance problems at CR entry may help identify patients who need a tailored assessment. Future research should include prospective, longitudinal studies to clarify causal pathways and evaluate whether early, tailored muscle-strengthening interventions can improve ETT participation rates and long-term clinical outcomes in patients with low muscle mass and musculoskeletal pain.

## 5. Conclusions

In patients referred for CR after myocardial infarction, musculoskeletal pain was highly prevalent and was associated with impaired functional status, balance problems, and lower appendicular skeletal muscle mass. However, after adjustment for clinical covariates, pain itself was not independently associated with initial ETT participation or VO_2_ peak, regardless of how pain intensity was categorized. In contrast, low SMI scores were independently associated with reduced ETT participation and showed only a borderline association with VO_2_ peak. These cross-sectional findings suggest that low muscle mass may be a relevant barrier to engagement in initial exercise testing; prospective studies are needed to determine whether early assessment of muscle mass and targeted interventions improve participation and functional recovery.

## Figures and Tables

**Figure 1:**
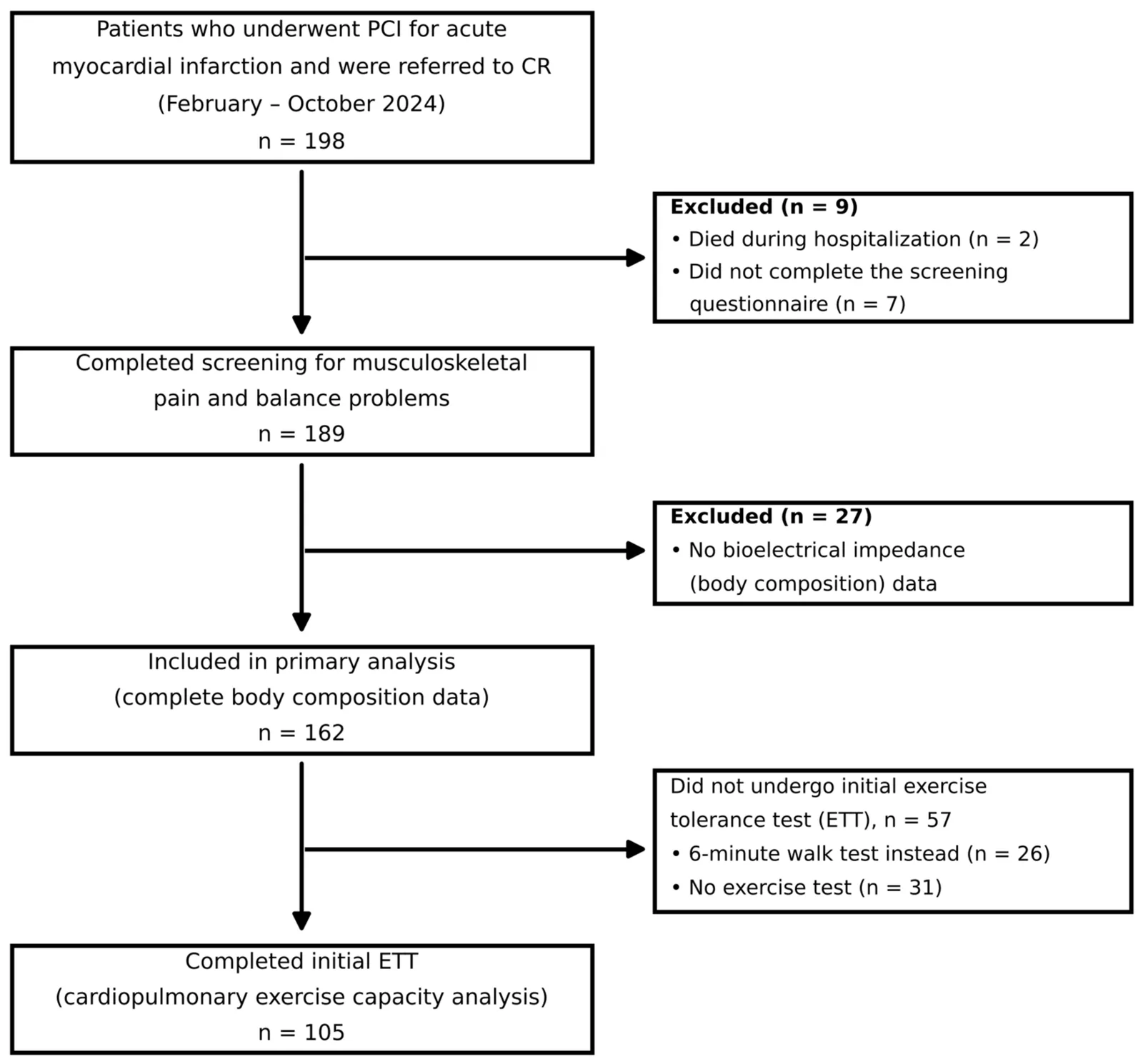
Flow diagram of patient inclusion and analysis. Of 198 patients who underwent percutaneous coronary intervention for acute myocardial infarction (February–October 2024), 9 were excluded (2 died during hospitalization and 7 did not complete the screening questionnaire), leaving 189 patients who were screened for musculoskeletal pain and balance problems. A further 27 patients without bioelectrical impedance data were excluded, leaving 162 patients for the primary analysis. Of these, 105 completed a symptom-limited exercise tolerance test and were included in the cardiopulmonary exercise capacity analysis, whereas 57 did not (26 underwent a 6-min walk test instead and 31 underwent no exercise test). CR, cardiac rehabilitation; ETT, exercise tolerance test; PCI, percutaneous coronary intervention.

**Table 1:** Baseline characteristics by sex (Total *n* = 162).

Variable	Men (*n* = 135)	Women (*n* = 27)	*p*-Value
Age (years)	64.9 ± 10.0	71.2 ± 12.6	0.005 *
pre-BMI (kg/m^2^)	24.43 ± 2.76	24.34 ± 3.38	0.459
ASM (kg)	23.49 ± 3.72	15.70 ± 2.39	<0.001 *
SMI (kg/m^2^)	8.23 ± 1.01	6.58 ± 0.75	<0.001 *
Education level (years)	11.44 ± 4.17	8.56 ± 3.33	<0.001 *
Smoking (Yes/No)	71/64	4/22	<0.001 *
Beta-blocker at discharge (Yes/No)	97/38	19/8	0.876
Statin at discharge (Yes/No)	129/6	25/2	0.621
Peak troponin T (ng/L), median [IQR]	2120 [600–5640]	906 [272–2005]	0.002 *
Multivessel disease (Yes/No)	57/77	11/16	0.863
Place of residence (metropolitan city/other city/rural)	1/84/50	1/15/11	0.302
Region of residence (Iksan/other Jeonbuk/outside Jeonbuk)	61/47/27	14/8/5	0.811

Data are presented as mean ± standard deviation, median [interquartile range], or *n*; smoking data were available for 161 patients, education data for 160 patients, and coronary angiographic data for 161 patients. Discharge medications, peak troponin T, the number of diseased vessels (multivessel disease, ≥2 vessels), and place of residence were abstracted from the medical records. Place of residence was classified from the residential address as a metropolitan city (Seoul or one of the metropolitan cities), another city (a dong, i.e., urban neighborhood, of a city), or rural (an eup or myeon [town or township] or a county [gun]); Iksan is the city in which the hospital is located, and Jeonbuk denotes Jeollabuk-do Province. Age was compared with the independent-samples *t*-test; pre-BMI, ASM, SMI, education level, and peak troponin T (non-normally distributed) with the Mann–Whitney U test; smoking, beta-blocker use, multivessel disease, and region with the chi-square test; and statin use and place of residence with Fisher’s exact or Fisher–Freeman–Halton exact tests. * *p* < 0.05. Abbreviations: pre-BMI, body mass index measured before the exercise tolerance test; ASM, appendicular skeletal muscle mass; SMI, skeletal muscle index.

**Table 2:** Patient distribution according to the musculoskeletal limitations screening questionnaire.

	*n* (162)	Men (135)	Women (27)	*p*-Value
Intensity of muscle/joint pain				0.010 *
No pain	71 (43.8)	66 (48.9)	5 (18.5)	
Pain rated 1–5	64 (39.5)	48 (35.6)	16 (59.3)	
Pain rated 6–10	27 (16.7)	21 (15.6)	6 (22.2)	
Effect of pain on ability to perform moderate exercise				0.072
No limitation	86 (53.1)	76 (56.3)	10 (37.0)	
Slight limitation	46 (28.4)	37 (27.4)	9 (33.3)	
Moderate limitation	21 (13.0)	17 (12.6)	4 (14.8)	
Severe limitation	9 (5.6)	5 (3.7)	4 (14.8)	
Pain relief with medications				0.043 *
No pain medication	126 (77.8)	109 (80.7)	17 (63.0)	
Pain medications present	36 (22.2)	26 (19.3)	10 (37.0)	
Degree of pain relief (among those on pain medication, *n* = 36)				0.584
No pain relief	4 (11.1)	4 (15.4)	0 (0.0)	
Slight pain relief	20 (55.6)	14 (53.8)	6 (60.0)	
Near-total pain relief	12 (33.3)	8 (30.8)	4 (40.0)	
Balance problems affecting ability to perform moderate exercise				0.871
No balance problems	122 (75.3)	102 (75.6)	20 (74.1)	
Balance problems present	40 (24.7)	33 (24.4)	7 (25.9)	
Degree of limitation (among those with balance problems, *n* = 40)				0.128
Slight limitation	32 (80.0)	28 (84.8)	4 (57.1)	
Moderate limitation	4 (10.0)	2 (6.1)	2 (28.6)	
Severe limitation	4 (10.0)	3 (9.1)	1 (14.3)	
Previously diagnosed chronic musculoskeletal disorder	66 (40.7)	54 (40.0)	12 (44.4)	0.668

Data are presented as *n* (%). *p*-values compare men and women using the chi-square test or, when any expected cell count was below 5, the Fisher–Freeman–Halton exact test. * *p* < 0.05.

**Table 3:** Clinical and demographic characteristics by pain status.

Variable	VAS 0 (*n* = 71)	VAS 1–10 (*n* = 91)	*p*-Value
Age (years)	65.14 ± 10.92	66.56 ± 10.54	0.404
LVEF (%)	47.76 ± 9.35	50.42 ± 8.86	0.066
BMI (kg/m^2^)	24.43 ± 2.57	24.41 ± 3.09	0.981
SMI (kg/m^2^)	8.10 ± 1.01	7.84 ± 1.23	0.141
ASM (kg)	23.29 ± 4.32	21.33 ± 4.61	0.022 *
KASI	64.14 ± 19.07	57.60 ± 21.05	0.027 *
Education level (years)	11.51 ± 4.00	10.52 ± 4.28	0.109
Sex (Men/Women)	66/5	69/22	0.004 *
STEMI/NSTEMI	33/38	49/42	0.352
Smoking (Yes/No)	47/24	28/62	<0.001 *
Hypertension (Yes/No)	53/18	56/35	0.078
Diabetes mellitus (Yes/No)	43/28	21/70	<0.001 *
Beta-blocker at discharge, yes (%)	53 (74.6)	63 (69.2)	0.448
Peak troponin T (ng/L), median [IQR]	2270 [527–5305]	1590 [497–4275]	0.336
Multivessel disease, yes (%)	29 (41.4)	39 (42.9)	0.856
Balance problem, yes (%)	9 (12.7)	31 (34.1)	0.002 *
Exercise limitation, yes (%)	13 (18.3)	63 (69.2)	<0.001 *
Chronic musculoskeletal disorder, yes (%)	18 (25.4)	48 (52.7)	<0.001 *

Data are presented as mean ± standard deviation, median [interquartile range], or *n* (%); smoking data were available for 161 patients, education data for 160 patients, and coronary angiographic data for 161 patients. Beta-blocker use at discharge, peak troponin T, and the number of diseased vessels (multivessel disease, ≥2 vessels) were abstracted from the medical records. Normally distributed variables were compared with the independent-samples *t*-test and non-normally distributed variables (ASM, KASI, education level, and peak troponin T) with the Mann–Whitney U test; categorical variables were compared with the chi-square test. * *p* < 0.05. Abbreviations: VAS, visual analog scale; LVEF, left ventricular ejection fraction; BMI, body mass index; SMI, skeletal muscle index; ASM, appendicular skeletal muscle mass; KASI, Korean Activity Scale/Index; STEMI, ST-segment elevation myocardial infarction; NSTEMI, non–ST-segment elevation myocardial infarction.

**Table 4:** Comparison of cardiopulmonary exercise capacity at the initial exercise tolerance test by pain status.

Variable	VAS 0 (*n* = 45)	VAS 1–10 (*n* = 60)	*p*-Value
KASI	68.35 ± 15.57	63.54 ± 18.28	0.136
Exercise time (sec)	766.42 ± 173.68	701.50 ± 185.85	0.017 *
RPE	13.93 ± 2.84	14.32 ± 1.64	0.821
METs (Q)	8.42 ± 2.35	7.36 ± 2.60	0.021 *
METs (M)	6.50 ± 1.86	5.97 ± 1.76	0.139
VO_2_ peak (mL/kg/min)	22.77 ± 6.50	20.74 ± 6.23	0.108
VE_max_ (L/min)	56.85 ± 17.89	48.19 ± 21.69	0.032 *
RER	1.07 ± 0.09	1.03 ± 0.11	0.044 *
ASM (kg)	23.36 ± 3.82	22.53 ± 4.26	0.303
SMI (kg/m^2^)	8.24 ± 1.01	8.17 ± 1.17	0.765
BMI (kg/m^2^)	24.42 ± 2.86	24.52 ± 2.90	0.865
Age (years)	62.67 ± 10.27	63.42 ± 9.18	0.693

Data are presented as mean ± standard deviation. Normally distributed variables (METs (M), VO_2_ peak, VEmax, RER, ASM, BMI, and age) were compared with the independent-samples *t*-test and non-normally distributed variables (KASI, exercise time, RPE, METs (Q), and SMI) with the Mann–Whitney U test. RPE, METs (Q), and METs (M) were available for 104 patients. * *p* < 0.05. Abbreviations: VAS, visual analog scale; KASI, Korean Activity Scale/Index; RPE, rating of perceived exertion; METs, metabolic equivalents; METs (Q), METs estimated from treadmill workload (modified Bruce protocol); METs (M), METs derived from measured peak oxygen uptake (VO_2_/3.5), i.e., the metric-unit expression of VO_2_ peak; VO_2_ peak, peak oxygen consumption; VE_max_, maximal minute ventilation; RER, respiratory exchange ratio; ASM, appendicular skeletal muscle mass; SMI, skeletal muscle index; BMI, body mass index.

**Table 5:** Multivariable logistic regression for participation in the initial ETT (*n* = 162).

Variable	B	SE	Wald	OR (Exp[B])	95% CI	*p*-Value
Sex (female)	0.897	0.618	2.111	2.453	0.731–8.229	0.146
Age (per year)	−0.062	0.021	8.419	0.940	0.902–0.980	0.004 *
Musculoskeletal pain (VAS ≥ 1)	−0.048	0.438	0.012	0.953	0.404–2.249	0.913
Diabetes mellitus	−0.415	0.430	0.932	0.660	0.284–1.534	0.334
Hypertension	−1.488	0.487	9.325	0.226	0.087–0.587	0.002 *
Ejection fraction (EF)	−0.016	0.021	0.608	0.984	0.945–1.025	0.436
SMI (per 1 kg/m^2^)	0.585	0.235	6.173	1.795	1.131–2.847	0.013 *

The dependent variable was participation in the initial ETT (1 = participated, 0 = did not participate); OR > 1 indicates higher odds of participation. Variance inflation factors were all < 2. McFadden pseudo-R^2^ = 0.20. * *p* < 0.05. Abbreviations: ETT, exercise tolerance test; VAS, visual analog scale; EF, ejection fraction; SMI, skeletal muscle index; B, unstandardized coefficient; SE, standard error; OR, odds ratio; CI, confidence interval.

**Table 6:** Multivariable linear regression for VO_2_ peak (*n* = 105).

Variable	B	SE	β	t	*p*-Value
Sex (female)	−1.936	1.957	−0.103	−0.989	0.325
Age (per year)	−0.227	0.060	−0.342	−3.797	<0.001 *
Pain intensity (VAS 0–10)	−0.216	0.193	−0.099	−1.121	0.265
Ejection fraction (EF)	0.085	0.062	0.121	1.378	0.171
SMI (kg/m^2^)	1.125	0.602	0.193	1.869	0.065

R^2^ = 0.268, adjusted R^2^ = 0.231. Variance inflation factors were all < 2. * *p* < 0.05. Abbreviations: B, unstandardized coefficient; SE, standard error; β, standardized coefficient; VAS, visual analog scale; EF, ejection fraction; SMI, skeletal muscle index.

## Data Availability

The data presented in this study are available on request from the corresponding author because of institutional privacy restrictions.

## Supplementary Materials

The following supporting information can be downloaded at: https://www.mdpi.com/article/10.3390/jcm15207951/s1. Table S1: Clinical characteristics according to pain category (VAS 0, 1–5, and 6–10); Table S2: Exercise capacity at the initial exercise tolerance test according to pain category; Table S3: Sensitivity analyses of the logistic and linear regression models; Table S4: Characteristics of initial exercise tolerance test participants and non-participants.

## References

[1] Visseren F.L.J., Mach F., Smulders Y.M., Carballo D., Koskinas K.C., Bäck M., Benetos A., Biffi A., Boavida J.M., Capodanno D. (2021). 2021 ESC Guidelines on cardiovascular disease prevention in clinical practice. Eur. Heart J..

[2] Virani S.S., Newby L.K., Arnold S.V., Bittner V., Brewer L.C., Demeter S.H., Dixon D.L., Fearon W.F., Hess B., Johnson H.M. (2023). 2023 AHA/ACC/ACCP/ASPC/NLA/PCNA guideline for the management of patients with chronic coronary disease: A report of the American Heart Association/American College of Cardiology Joint Committee on Clinical Practice Guidelines. Circulation.

[3] Mathews L., Brewer L.C. (2021). A review of disparities in cardiac rehabilitation: Evidence, drivers, and solutions. J. Cardiopulm. Rehabil. Prev..

[4] Grace S.L., Taylor R.S., Gaalema D.E., Redfern J., Kotseva K., Ghisi G. (2023). Cardiac rehabilitation: A global perspective on where we have come and where we must go. JACC Adv..

[5] Foster E.J., Munoz S.A., Crabtree D., Leslie S.J., Gorely T. (2021). Barriers and facilitators to participating in cardiac rehabilitation and physical activity in a remote and rural population: A cross-sectional survey. Cardiol. J..

[6] Ruano-Ravina A., Pena-Gil C., Abu-Assi E., Raposeiras S., van ’t Hof A., Meindersma E., Bossano Prescott E.I., González-Juanatey J.R. (2016). Participation and adherence to cardiac rehabilitation programs. A systematic review. Int. J. Cardiol..

[7] Kim S.H., Ro J.S., Kim Y., Leigh J.H., Kim W.S. (2020). Underutilization of hospital-based cardiac rehabilitation after acute myocardial infarction in Korea. J. Korean Med. Sci..

[8] Choi H.E., Kim C., Lee D.J., Joo J.E., Kim H.S. (2023). Participation and prognostic impact of cardiac rehabilitation after acute coronary syndrome: Big-data study of the Korean National Health Insurance Service. J. Korean Med. Sci..

[9] Khan M., Neuhof A., Tsai S., Powell S., Silaj E., MacNeill H., Oh P., Cott C. (2014). Examination of cardiac rehabilitation participants referred to a musculoskeletal clinic. J. Cardiopulm. Rehabil. Prev..

[10] Goel K., Shen J., Wolter A.D., Beck K.M., Leth S.E., Thomas R.J., Squires R.W., Perez-Terzic C.M. (2010). Prevalence of musculoskeletal and balance disorders in patients enrolled in phase II cardiac rehabilitation. J. Cardiopulm. Rehabil. Prev..

[11] Supervia M., Medina-Inojosa J.R., Pérez-Terzic C.M., Sharma S., Goel K., Vickers Douglas K., Salz K., Thomas R.J. (2021). Impact of musculoskeletal limitations on cardiac rehabilitation participation. Front. Cardiovasc. Med..

[12] Cruz-Jentoft A.J., Bahat G., Bauer J., Boirie Y., Bruyère O., Cederholm T., Cooper C., Landi F., Rolland Y., Sayer A.A. (2019). Sarcopenia: Revised European consensus on definition and diagnosis. Age Ageing.

[13] Erkan M., Zengin İ., Bekircavuşoğlu S., Topal D., Bulut T., Erkan H. (2024). Effect of sarcopenia on coronary atherosclerotic burden, lesion complexity, and major cardiovascular events in elderly patients with acute coronary syndrome: A 1-year follow-up study. Angiology.

[14] Fülster S., Tacke M., Sandek A., Ebner N., Tschöpe C., Doehner W., Anker S.D., von Haehling S. (2013). Muscle wasting in patients with chronic heart failure: Results from the studies investigating co-morbidities aggravating heart failure (SICA-HF). Eur. Heart J..

[15] Sung J., On Y.K., Kim H.S., Chae I.H., Sohn D.W., Oh B.H., Lee M.M., Park Y.B., Choi Y.S., Lee Y.W. (2000). Development of Korean Activity Scale/Index (KASI). Korean Circ. J..

[16] Hlatky M.A., Boineau R.E., Higginbotham M.B., Lee K.L., Mark D.B., Califf R.M., Cobb F.R., Pryor D.B. (1989). A brief self-administered questionnaire to determine functional capacity (the Duke Activity Status Index). Am. J. Cardiol..

[17] Grace S.L., Shanmugasegaram S., Gravely-Witte S., Brual J., Suskin N., Stewart D.E. (2009). Barriers to cardiac rehabilitation: Does age make a difference?. J. Cardiopulm. Rehabil. Prev..

[18] Vlaeyen J.W.S., Crombez G., Linton S.J. (2016). The fear-avoidance model of pain. Pain.

[19] Geneen L.J., Moore R.A., Clarke C., Martin D., Colvin L.A., Smith B.H. (2017). Physical activity and exercise for chronic pain in adults: An overview of Cochrane Reviews. Cochrane Database Syst. Rev..

[20] American Association of Cardiovascular and Pulmonary Rehabilitation (2021). Guidelines for Cardiac Rehabilitation Programs.

[21] Dores H., Mendes M., Abreu A., Durazzo A., Rodrigues C., Vilela E., Cunha G., Gomes Pereira J., Bento L., Moreno L. (2024). Cardiopulmonary exercise testing in clinical practice: Principles, applications, and basic interpretation. Rev. Port. Cardiol..

[22] Kornuijt A., Bongers B.C., Marcellis R.G.J., Lenssen A.F. (2024). Submaximal cardiopulmonary exercise testing to assess preoperative aerobic capacity in patients with knee osteoarthritis scheduled for total knee arthroplasty: A feasibility study. Physiother. Theory Pract..

[23] Daly J., Sindone A.P., Thompson D.R., Hancock K., Chang E., Davidson P. (2002). Barriers to participation in and adherence to cardiac rehabilitation programs: A critical literature review. Prog. Cardiovasc. Nurs..

[24] Damluji A.A., Alfaraidhy M., AlHajri N., Rohant N.N., Kumar M., Al Malouf C., Bahrainy S., Kwak M.J., Batchelor W.B., Forman D.E. (2023). Sarcopenia and cardiovascular diseases. Circulation.

[25] Fleg J.L., Morrell C.H., Bos A.G., Brant L.J., Talbot L.A., Wright J.G., Lakatta E.G. (2005). Accelerated longitudinal decline of aerobic capacity in healthy older adults. Circulation.

[26] Seals D.R., Jablonski K.L., Donato A.J. (2011). Aging and vascular endothelial function in humans. Clin. Sci..

[27] Betik A.C., Hepple R.T. (2008). Determinants of VO_2 max_ decline with aging: An integrated perspective. Appl. Physiol. Nutr. Metab..

[28] Ross R., Blair S.N., Arena R., Church T.S., Després J.P., Franklin B.A., Haskell W.L., Kaminsky L.A., Levine B.D., Lavie C.J. (2016). Importance of assessing cardiorespiratory fitness in clinical practice: A case for fitness as a clinical vital sign: A scientific statement from the American Heart Association. Circulation.

[29] Larsson C., Ekvall Hansson E., Sundquist K., Jakobsson U. (2016). Impact of pain characteristics and fear-avoidance beliefs on physical activity levels among older adults with chronic pain: A population-based, longitudinal study. BMC Geriatr..

[30] Cohen S.P., Vase L., Hooten W.M. (2021). Chronic pain: An update on burden, best practices, and new advances. Lancet.

[31] Landi F., Cruz-Jentoft A.J., Liperoti R., Russo A., Giovannini S., Tosato M., Capoluongo E., Bernabei R., Onder G. (2013). Sarcopenia and mortality risk in frail older persons aged 80 years and older: Results from ilSIRENTE study. Age Ageing.

[32] Bekfani T., Bekhite Elsaied M., Derlien S., Nisser J., Westermann M., Nietzsche S., Hamadanchi A., Fröb E., Westphal J., Haase D. (2020). Skeletal muscle function, structure, and metabolism in patients with heart failure with reduced ejection fraction and heart failure with preserved ejection fraction. Circ. Heart Fail..

[33] Kitzman D.W., Nicklas B., Kraus W.E., Lyles M.F., Eggebeen J., Morgan T.M., Haykowsky M. (2014). Skeletal muscle abnormalities and exercise intolerance in older patients with heart failure and preserved ejection fraction. Am. J. Physiol. Heart Circ. Physiol..

[34] Gielen S., Adams V., Möbius-Winkler S., Linke A., Erbs S., Yu J., Kempf W., Schubert A., Schuler G., Hambrecht R. (2003). Anti-inflammatory effects of exercise training in the skeletal muscle of patients with chronic heart failure. J. Am. Coll. Cardiol..

[35] MacEachern E., Quach J., Giacomantonio N., Theou O., Hillier T., Abel-Adegbite I., Gonzalez-Lara M., Kehler D.S. (2024). Cardiac rehabilitation and frailty: A systematic review and meta-analysis. Eur. J. Prev. Cardiol..

[36] Schopfer D.W., Forman D.E. (2016). Cardiac rehabilitation in older adults. Can. J. Cardiol..

[37] Choi S.B., Kim J.H. (2023). Relationship between number of cardiac rehabilitation exercise training sessions, muscle mass, and cardiorespiratory fitness in rural elderly patients with coronary artery disease. J. Multidiscip. Healthc..

[38] Rolland Y., Czerwinski S., Abellan Van Kan G., Morley J.E., Cesari M., Onder G., Woo J., Baumgartner R., Pillard F., Boirie Y. (2008). Sarcopenia: Its assessment, etiology, pathogenesis, consequences and future perspectives. J. Nutr. Health Aging.

[39] Beaudart C., Dawson A., Shaw S.C., Harvey N.C., Kanis J.A., Binkley N., Reginster J.Y., Chapurlat R., Chan D.C., Bruyère O. (2017). Nutrition and physical activity in the prevention and treatment of sarcopenia: Systematic review. Osteoporos. Int..

[40] Cesari M., Landi F., Vellas B., Bernabei R., Marzetti E. (2014). Sarcopenia and physical frailty: Two sides of the same coin. Front. Aging Neurosci..

[41] Dibben G.O., Faulkner J., Oldridge N., Rees K., Thompson D.R., Zwisler A.D., Taylor R.S. (2023). Exercise-based cardiac rehabilitation for coronary heart disease: A meta-analysis. Eur. Heart J..

[42] Ambrosetti M., Abreu A., Corrà U., Davos C.H., Hansen D., Frederix I., Iliou M.C., Pedretti R.F.E., Schmid J.P., Vigorito C. (2021). Secondary prevention through comprehensive cardiovascular rehabilitation: From knowledge to implementation. 2020 update. A position paper from the Secondary Prevention and Rehabilitation Section of the European Association of Preventive Cardiology. Eur. J. Prev. Cardiol..

[43] Paluch A.E., Boyer W.R., Franklin B.A., Laddu D., Lobelo F., Lee D.C., McDermott M.M., Swift D.L., Webel A.R., Lane A. (2024). Resistance exercise training in individuals with and without cardiovascular disease: 2023 update: A scientific statement from the American Heart Association. Circulation.

[44] Tanriverdi A., Ozcan Kahraman B., Ozsoy I., Acar S., Senturk B., Ozpelit E., Akdeniz B., Savci S. (2020). Balance performance in patients with heart failure. Heart Lung.

