# Supplementary material for: Pain, Low Muscle Mass, and Exercise Participation After Myocardial Infarction: Determinants of Initial Exercise Testing at a Tertiary Center Serving a Rural Region
Source: J Clin Med. 2026 Oct 14;15(20):7951. doi: 10.3390/jcm15207951 (PMC13706407; doi:10.3390/jcm15207951)
Supplement: Supplementary file 1 [file jcm-15-07951-s001.zip › jcm-4583851-supplementary.pdf]

Supplementary Materials

Pain, Low Muscle Mass, and Exercise Participation After Myocardial Infarction: Determinants of Initial Exercise Testing at a Tertiary Center Serving a Rural Region

Table S1. Clinical characteristics according to pain category (VAS 0, 1–5, and 6–10; n = 162)

Variable	VAS 0 (n = 71)	VAS 1–5 (n = 64)	VAS 6–10 (n = 27)	P-value
Age (years)	65.1 ± 10.9	66.0 ± 9.7	67.9 ± 12.4	0.526[a]
LVEF (%)	47.8 ± 9.3	50.1 ± 8.6	51.1 ± 9.7	0.166[a]
BMI (kg/m²)	24.4 ± 2.6	24.4 ± 2.9	24.4 ± 3.4	0.996[a]
SMI (kg/m²)	8.10 ± 1.01	7.75 ± 1.06	8.06 ± 1.58	0.177[a]
ASM (kg)	23.3 ± 4.3	21.1 ± 4.4	21.9 ± 5.2	0.037*[b]
KASI	64.1 ± 19.1	58.2 ± 19.6	56.1 ± 24.6	0.085[b]
Education level (years)	11.5 ± 4.0	10.7 ± 4.2	10.1 ± 4.5	0.240[b]
Female sex, n (%)	5 (7.0)	16 (25.0)	6 (22.2)	0.010*[f]
STEMI, n (%)	33 (46.5)	33 (51.6)	16 (59.3)	0.518[c]
Hypertension, n (%)	53 (74.6)	46 (71.9)	10 (37.0)	0.001*[c]
Diabetes mellitus, n (%)	43 (60.6)	10 (15.6)	11 (40.7)	<0.001*[c]
Smoking, n (%)	47 (66.2)	17 (26.6)	11 (42.3)	<0.001*[c]
Balance problem, n (%)	9 (12.7)	14 (21.9)	17 (63.0)	<0.001*[c]
Exercise limitation due to pain, n (%)	13 (18.3)	43 (67.2)	20 (74.1)	<0.001*[c]
Pain medication, n (%)	0 (0.0)	21 (32.8)	15 (55.6)	<0.001*[c]
Chronic musculoskeletal disorder, n (%)	18 (25.4)	23 (35.9)	25 (92.6)	<0.001*[c]
Initial ETT participation, n (%)	45 (63.4)	43 (67.2)	17 (63.0)	0.877[c]

Data are presented as mean ± standard deviation or n (%). Smoking data were available for 161 patients and education data for 160 patients. Tests: [a] one-way ANOVA; [b] Kruskal–Wallis test; [c] chi-square test; [f] Fisher–Freeman–Halton exact test. * P < 0.05.

Abbreviations: VAS, visual analog scale; LVEF, left ventricular ejection fraction; BMI, body mass index; SMI, skeletal muscle index; ASM, appendicular skeletal muscle mass; KASI, Korean Activity Scale/Index; STEMI, ST-segment elevation myocardial infarction; ETT, exercise tolerance test.

Table S2. Exercise capacity at the initial exercise tolerance test according to pain category (n = 105)

Variable	VAS 0 (n = 45)	VAS 1–5 (n = 43)	VAS 6–10 (n = 17)	P-value
KASI	68.3 ± 15.6	62.7 ± 17.5	65.7 ± 20.5	0.152[b]
Exercise time (s)	766 ± 174	699 ± 190	707 ± 180	0.056[b]
RPE	13.9 ± 2.8	14.3 ± 1.7	14.5 ± 1.6	0.658[b]
METs (Q)	8.43 ± 2.35	7.46 ± 2.72	7.09 ± 2.35	0.066[b]
METs (M)	6.51 ± 1.86	5.93 ± 1.74	6.07 ± 1.85	0.320[a]
VO_2 peak (mL/kg/min)	22.8 ± 6.5	20.5 ± 6.2	21.2 ± 6.5	0.255[a]
VEmax (L/min)	56.8 ± 17.9	48.6 ± 21.3	47.2 ± 23.3	0.097[a]
RER	1.07 ± 0.09	1.02 ± 0.10	1.04 ± 0.13	0.107[a]
SMI (kg/m^2)	8.24 ± 1.01	7.99 ± 1.05	8.61 ± 1.37	0.285[b]
Age (years)	62.7 ± 10.3	63.7 ± 8.9	62.8 ± 10.1	0.878[a]

Data are presented as mean ± standard deviation. RPE, METs (Q), and METs (M) were available for 104 patients. Tests: [a] one-way ANOVA; [b] Kruskal–Wallis test. * $P < 0.05$.

Abbreviations: VAS, visual analog scale; KASI, Korean Activity Scale/Index; RPE, rating of perceived exertion; METs (Q), metabolic equivalents estimated from treadmill workload; METs (M), metabolic equivalents derived from measured VO_2 peak; VEmax, maximal minute ventilation; RER, respiratory exchange ratio; SMI, skeletal muscle index.

Table S3. Sensitivity analyses of the logistic and linear regression models

Model	SMI (per 1 kg/m²)	Age (per year)	Hypertension	Pain	Added variable(s)
A. Logistic regression for initial ETT participation: OR (95% CI); p					
Primary model (Table 5)	1.79 (1.13–2.85); 0.013	0.94 (0.90–0.98); 0.004	0.23 (0.09–0.59); 0.002	0.95 (0.40–2.25); 0.913	—
Pain as continuous VAS (per point)	1.81 (1.14–2.86); 0.012	0.94 (0.90–0.98); 0.005	0.20 (0.07–0.55); 0.002	0.94 (0.81–1.10); 0.446	—
Pain in three categories	1.81 (1.14–2.87); 0.012	0.94 (0.90–0.98); 0.006	0.19 (0.07–0.53); 0.002	1–5: 1.14; 0.786 / 6–10: 0.62; 0.442	—
+ Balance problem	1.80 (1.13–2.86); 0.013	0.94 (0.90–0.98); 0.005	0.22 (0.08–0.57); 0.002	1.03 (0.43–2.52); 0.940	0.73 (0.29–1.79); 0.485
+ Chronic musculoskeletal disorder	1.78 (1.12–2.82); 0.015	0.94 (0.90–0.98); 0.004	0.21 (0.08–0.56); 0.002	1.05 (0.42–2.59); 0.919	0.76 (0.34–1.74); 0.521
+ Education level (per year) (n = 160)	1.77 (1.12–2.81); 0.015	0.95 (0.91–0.99); 0.028	0.22 (0.09–0.58); 0.002	0.98 (0.41–2.32); 0.956	1.05 (0.95–1.17); 0.335
+ All three variables (n = 160)	1.76 (1.11–2.79); 0.017	0.95 (0.91–1.00); 0.034	0.21 (0.08–0.55); 0.002	1.13 (0.45–2.83); 0.802	Bal: 0.84; 0.710 / MSK: 0.75; 0.510 / Edu: 1.05; 0.357
+ Beta-blocker at discharge	1.80 (1.13–2.87); 0.014	0.94 (0.90–0.98); 0.003	0.24 (0.09–0.61); 0.003	0.97 (0.41–2.31); 0.951	0.60 (0.25–1.43); 0.245
+ Peak troponin T (log_{10})	1.80 (1.14–2.86); 0.012	0.94 (0.90–0.98); 0.005	0.21 (0.08–0.56); 0.002	0.94 (0.40–2.24); 0.894	0.74 (0.39–1.41); 0.363
+ Multivessel disease (n = 161)	1.77 (1.11–2.81); 0.017	0.94 (0.90–0.98); 0.004	0.22 (0.08–0.59); 0.002	0.96 (0.41–2.26); 0.920	1.09 (0.50–2.37); 0.834
+ Rural residence	1.80 (1.13–2.87); 0.014	0.94 (0.90–0.98); 0.005	0.22 (0.08–0.57); 0.002	0.98 (0.41–2.32); 0.957	0.54 (0.25–1.18); 0.121
+ All four clinical/residence variables (n = 161)	1.79 (1.11–2.87); 0.017	0.94 (0.90–0.98); 0.005	0.21 (0.08–0.58); 0.002	0.98 (0.41–2.36); 0.972	BB: 0.63; 0.305 / Trop: 0.74; 0.355 / MVD: 1.04; 0.921 / Rural: 0.57; 0.162
Restricted to body composition measured during admission (n = 155)	1.66 (1.03–2.66); 0.036	0.94 (0.90–0.98); 0.004	0.23 (0.09–0.62); 0.003	0.94 (0.40–2.24); 0.891	—

(continued on next page)

Table S3. (continued)

Model	SMI (per 1 kg/m²)	Age (per year)	Hypertension	Pain	Added variable(s)
B. Linear regression for VO_2 peak: B (95% CI); p					
Primary model (Table 6)	1.12 (−0.07 to 2.32); 0.065	−0.23 (−0.35 to −0.11); <0.001	—	−0.22 (−0.60 to 0.17); 0.265	—
Pain in three categories	1.08 (−0.12 to 2.28); 0.078	−0.22 (−0.34 to −0.11); <0.001	—	1–5: −1.76; 0.151 / 6–10: −2.05; 0.208	—
+ Balance problem	1.14 (−0.08 to 2.35); 0.066	−0.23 (−0.35 to −0.11); <0.001	—	−0.23 (−0.65 to 0.19); 0.284	0.18 (−2.67 to 3.03); 0.899
+ Chronic musculoskeletal disorder	1.05 (−0.13 to 2.23); 0.081	−0.23 (−0.34 to −0.11); <0.001	—	−0.06 (−0.47 to 0.34); 0.752	−2.37 (−4.75 to 0.01); 0.051
+ Education level (per year)	1.13 (−0.07 to 2.33); 0.065	−0.24 (−0.37 to −0.11); <0.001	—	−0.23 (−0.63 to 0.16); 0.240	−0.07 (−0.40 to 0.25); 0.657
+ All three variables	1.06 (−0.14 to 2.26); 0.083	−0.24 (−0.37 to −0.11); <0.001	—	−0.09 (−0.54 to 0.35); 0.673	Bal: 0.18; 0.901 / MSK: −2.42; 0.049 / Edu: −0.09; 0.568
+ Beta-blocker at discharge	1.09 (−0.11 to 2.29); 0.074	−0.22 (−0.34 to −0.11); <0.001	—	−0.21 (−0.59 to 0.17); 0.281	1.18 (−1.37 to 3.73); 0.361
+ Peak troponin T (log_{10})	1.13 (−0.07 to 2.33); 0.065	−0.23 (−0.35 to −0.11); <0.001	—	−0.22 (−0.60 to 0.17); 0.267	−0.13 (−2.03 to 1.76); 0.889
+ Multivessel disease (n = 104)	0.92 (−0.30 to 2.14); 0.139	−0.22 (−0.34 to −0.10); <0.001	—	−0.19 (−0.58 to 0.19); 0.319	0.60 (−1.66 to 2.86); 0.601
+ Rural residence	1.20 (0.00 to 2.40); 0.049	−0.23 (−0.35 to −0.11); <0.001	—	−0.19 (−0.58 to 0.19); 0.319	−1.39 (−3.76 to 0.98); 0.246
+ All four clinical/residence variables (n = 104)	0.94 (−0.30 to 2.18); 0.136	−0.22 (−0.35 to −0.10); <0.001	—	−0.16 (−0.55 to 0.22); 0.402	BB: 1.66; 0.215 / Trop: 0.04; 0.971 / MVD: 0.84; 0.479 / Rural: −1.48; 0.228
Restricted to body composition measured during admission (n = 99)	1.49 (0.13 to 2.84); 0.032	−0.21 (−0.33 to −0.09); <0.001	—	−0.31 (−0.73 to 0.11); 0.148	—

All logistic models were adjusted for sex, age, pain, diabetes mellitus, hypertension, LVEF, and SMI (n = 162 unless stated otherwise; 160 when education was included and 161 when multivessel disease was included); all linear models were adjusted for sex, age, pain intensity, LVEF, and SMI (n = 105 unless stated otherwise; 104 when multivessel disease was included). Hypertension was not included in the linear models. Beta-blocker use at discharge, peak troponin T, the number of diseased vessels, and place of residence were obtained from the medical records; rural residence was defined as residence in an eup or myeon (town or township) or a county (gun). The restricted models include only patients whose body composition was measured during the index admission. Variance inflation factors were below 2 in all primary and single-addition models.

Abbreviations: ETT, exercise tolerance test; OR, odds ratio; CI, confidence interval; B, unstandardized coefficient; SMI, skeletal muscle index; VAS, visual analog scale; Bal, balance problem; MSK, chronic musculoskeletal disorder; Edu, education level (years); BB, beta-blocker at discharge; Trop, log_{10} peak troponin T; MVD, multivessel disease (≥ 2 diseased vessels); LVEF, left ventricular ejection fraction.

Table S4. Characteristics of initial exercise tolerance test participants and non-participants (n = 162)

Variable	Participants (n = 105)	Non-participants (n = 57)	P-value	6MWT only (n = 26)	No exercise test (n = 31)
Age (years)	63.1 ± 9.6	71.2 ± 10.7	<0.001*[c]	75.1 ± 8.3	67.9 ± 11.4
LVEF (%)	49.4 ± 9.1	49.1 ± 9.3	0.429[d]	49.3 ± 11.2	48.8 ± 7.6
BMI (kg/m²)	24.5 ± 2.8	24.3 ± 2.9	0.605[d]	23.7 ± 3.0	24.8 ± 2.7
SMI (kg/m²)	8.20 ± 1.10	7.51 ± 1.10	<0.001*[d]	7.08 ± 1.14	7.86 ± 0.94
ASM (kg)	22.9 ± 4.1	20.9 ± 5.2	0.002*[d]	19.1 ± 5.6	22.4 ± 4.3
KASI	65.6 ± 17.3	51.0 ± 22.4	<0.001*[d]	45.8 ± 22.1	55.4 ± 22.1
Education level (years)	11.6 ± 3.8	9.7 ± 4.6	0.005*[d]	8.3 ± 3.7	11.0 ± 5.1
VAS score	2.6 ± 2.9	2.5 ± 3.0	0.814[d]	3.4 ± 3.0	1.8 ± 2.8
Female sex, n (%)	14 (13.3)	13 (22.8)	0.122[e]	10 (38.5)	3 (9.7)
STEMI, n (%)	55 (52.4)	27 (47.4)	0.542[e]	12 (46.2)	15 (48.4)
Hypertension, n (%)	60 (57.1)	49 (86.0)	<0.001*[e]	22 (84.6)	27 (87.1)
Diabetes mellitus, n (%)	37 (35.2)	27 (47.4)	0.132[e]	11 (42.3)	16 (51.6)
Smoking, n (%)	52 (50.0)	23 (40.4)	0.240[e]	7 (26.9)	16 (51.6)
Pain (VAS ≥ 1), n (%)	60 (57.1)	31 (54.4)	0.736[e]	18 (69.2)	13 (41.9)
Exercise limitation due to pain, n (%)	48 (45.7)	28 (49.1)	0.678[e]	16 (61.5)	12 (38.7)
Balance problem, n (%)	24 (22.9)	16 (28.1)	0.462[e]	11 (42.3)	5 (16.1)
Pain medication, n (%)	24 (22.9)	12 (21.1)	0.792[e]	8 (30.8)	4 (12.9)
Chronic musculoskeletal disorder, n (%)	41 (39.0)	25 (43.9)	0.552[e]	15 (57.7)	10 (32.3)
Beta-blocker at discharge, n (%)	72 (68.6)	44 (77.2)	0.245[e]	19 (73.1)	25 (80.6)
Multivessel disease, n (%)	42 (40.4)	26 (45.6)	0.521[e]	14 (53.8)	12 (38.7)
Peak troponin T (ng/L), median [IQR]	1900 [486–4290]	2060 [508–5250]	0.614[d]	1890 [462–4848]	2570 [583–5520]
Pain category 0 / 1–5 / 6–10, n	45 / 43 / 17	26 / 21 / 10	0.877[e]	8 / 12 / 6	18 / 9 / 4
6-minute walk distance (m), median (IQR)	—	—		240 (126–360)	—
Documented reason for not undergoing the initial ETT, n					
Musculoskeletal pain or gait impairment				19	
Neurological or cognitive disorder				4	
Exertional symptoms (e.g., chest discomfort, dyspnea)				3	
Did not attend the outpatient assessment; no reason recorded					23
Distance or transfer to another hospital					3
Declined rehabilitation or testing					2
Poor general condition					2
Personal reasons					1

Data are presented as mean ± standard deviation or n (%). P-values compare participants with all non-participants; the two non-participant subgroups are shown descriptively. Tests: [c] t-test; [d] Mann–Whitney U test; [e] chi-square test. * $P < 0.05$.

Reasons for not undergoing the initial ETT, beta-blocker use at discharge, peak troponin T, and the number of diseased vessels (multivessel disease, ≥ 2 vessels; available for 161 patients) were abstracted retrospectively from the medical records; one reason was assigned per patient.

Abbreviations: ETT, exercise tolerance test; 6MWT, 6-minute walk test; LVEF, left ventricular ejection fraction; BMI, body mass index; SMI, skeletal muscle index; ASM, appendicular skeletal muscle mass; KASI, Korean Activity Scale/Index; VAS, visual analog scale; STEMI, ST-segment elevation myocardial infarction.
